# Neuropathological Mechanisms Associated with Pesticides in Alzheimer’s Disease

**DOI:** 10.3390/toxics8020021

**Published:** 2020-03-25

**Authors:** Bor Luen Tang

**Affiliations:** 1 Department of Biochemistry, Yong Loo Lin School of Medicine, National University of Singapore, Singapore 117596, Singapore; bchtbl@nus.edu.sg; Tel.: +65-6516-1040; 2 NUS Graduate School for Integrative Sciences and Engineering, National University of Singapore, Singapore 119077, Singapore

**Keywords:** Alzheimer’s disease, amyloid beta (Aβ), fungicide, Glycogen synthase kinase-3, pesticide, tau

## Abstract

Environmental toxicants have been implicated in neurodegenerative diseases, and pesticide exposure is a suspected environmental risk factor for Alzheimer’s disease (AD). Several epidemiological analyses have affirmed a link between pesticides and incidence of sporadic AD. Meanwhile, in vitro and animal models of AD have shed light on potential neuropathological mechanisms. In this paper, a perspective on neuropathological mechanisms underlying pesticides’ induction of AD is provided. Proposed mechanisms range from generic oxidative stress induction in neurons to more AD-specific processes involving amyloid-beta (Aβ) and hyperphosphorylated tau (p-tau). Mechanisms that are more speculative or indirect in nature, including somatic mutation, epigenetic modulation, impairment of adult neurogenesis, and microbiota dysbiosis, are also discussed. Chronic toxicity mechanisms of environmental pesticide exposure crosstalks in complex ways and could potentially be mutually enhancing, thus making the deciphering of simplistic causal relationships difficult.

## 1. Introduction

Alzheimer’s disease (AD) [1,2] is the most prevalent cause of age-associated dementia worldwide [3]. Late-onset AD is largely sporadic, with those harboring gene mutations recognized as causative of the familial form of the disease constituting less than 5% of all patients [1,4]. The brains of AD patients and animal models typically exhibit two characteristic pathological features. Intraneuronal neurofibrillary tangles (IFTs) are enriched in the hyperphosphorylated form of a microtubule-binding protein tau [5], while extracellular amyloid plaques consist of insoluble aggregates of amyloid β (Aβ) peptides generated via proteolytic processing of the amyloid precursor protein (APP) [6]. Typical amyloidogenic APP processing occurs by the sequential action of the classical β-secretase, the β-site APP cleaving enzyme 1 (BACE1) [7], and γ-secretase [8], which generate Aβ peptides, mainly Aβ1-40 and Aβ1-42. These, particularly the latter, are neurotoxic and aggregation-prone. Alternative β-secretases, including δ- and η-secretases, adds to the complexity of Aβ products [9]. On the other hand, initial cleavage by α-secretases such as A Disintegrin and Metalloproteinase 10 (ADAM10) [10] promotes what was termed non-amyloidogenic processing, which does not produce Aβ1-40 and Aβ1-42 [11].

The pathological features point to disease etiology. As such the amyloid cascade hypothesis [12,13] postulates that the production of Aβ1-42 (and related peptides) with the subsequent formation of amyloid plaques as the etiological origin of AD, while the amyloid-β oligomer hypothesis emphasizes the greater neurotoxicity of soluble Aβ oligomers compared to those within insoluble amyloid plaques [14]. In vivo, both tau and Aβ contribute to AD pathology [14,15] and the development and progress of the pathological features involving these two are highly intertwined [2,16]. Mutations in APP and the presenilins (PS) (which are the catalytic components of γ-secretase [17,18]) that underlie familial and early-onset AD [4,19,20] invariably cause an increase in Aβ production. Despite extensive advances in AD genetics and molecular pathology, definitive triggers or etiological origin for the late-onset, sporadic, or idiopathic form of AD prevalent in majority of patients has remained largely elusive. As in other sporadic late-onset neurodegenerative diseases, onset of sporadic AD could be broadly attributed to gene-environment interactions [21,22]. The Apolipoprotein E4 (APOE4) ε4 allele is a prevalent genetic risk factor for late-onset AD [23], but environmental factors are more varied and less well-defined in terms of pathological profile.

Environmental toxicants have been extensively linked to neurodegenerative disorders [24,25]. Homeostatic dysregulation of metals in brain cells and tissues or the accumulation of toxic metals have been linked to various neurodegenerative diseases, including AD [26,27]. Lead (Pb) is known to be neurotoxic and perturbs Aβ generation and clearance [28], and Pb exposure has been implicated in AD [29], but case-control studies have not nailed down a clear link between tissue Pb accumulation and AD [30]. Copper (Cu) affects Aβ aggregation kinetics [31] and AD patients have altered Cu metabolism [32]. Chronic exposure to aluminium (Al) has been shown to increase AD risk [33], but the notion remains controversial [34]. Organic neurotoxins have also been extensively linked to neurodegeneration, with 1-methyl-4-phenyl-1,2,3,6-tetrahydropyridine (MPTP) in Parkinson’s disease (PD) [35] and β-N-methylamino-L-alanine (BMAA) in the Amyotrophic Lateral Sclerosis/Parkinsonism Dementia Complex epidemically prevalent on the island of Guam [36] being perhaps the most prominent in the popular press.

Pesticides, including insecticides, herbicides, and fungicides, are a large and diverse group of organic environmental toxicants affecting neuronal health [25,37,38,39,40,41]. In the past 5 years, the link between pesticide exposure and AD has been considerably strengthened, while basic understanding of pesticide-associated neuropathology has improved. In this brief review, the links between pesticide exposure and AD are summarized and discussed. Information on disease etiology, particularly recently-obtained mechanistic insights from cellular and animal models, is highlighted.

## 2. Epidemiological Links between Pesticide Exposure and Alzheimer’s Disease

Epidemiological links between pesticide exposure and AD has been fairly strong [25,37,39,41], particularly for occupational exposure of organophosphates in men [39,41], but not without controversy [42]. Wartime exposure to Agent Orange is strongly associated with many ailments, including neurological disorders like AD (adjusted odds ratio (aOR): 95%; confidence interval (CI): 1.64, 1.12–2.41) [43]. In terms of lifetime environmental exposure, cross-sectional and prospective data from the Maastricht Aging Study found exposure to pesticides to increase the risk of mild cognitive impairment (Cross-sectional, self-reported aOR, 95% CI: 4.94, 1.53–16.1), usually viewed as prodromal to AD [44]. The Cache County study (3084 enrollees, 344 with AD) also indicated a significant risk of incident AD from occupational pesticide (a majority of which to organophosphates and organochlorines) exposure (hazard ratio (HR), 95% CI: 1.42, 1.06–1.91) [45]. An ecological study using averaged prevalence rates of AD in selected Andalusian health districts showed that the population living in areas with high pesticide use (17,429 cases, 2185 with AD) had an increased AD risk (OR, 95% CI: 2.1, 1.96–2.25) [46]. In a case-control study with AD patients and control participants in the USA, levels of 1,1-dichloro-2,2-bis(4-chlorophenyl)ethene (DDE),a metabolite of 1-chloro-4-[2,2,2-trichloro-1-(4-chlorophenyl)ethyl]benzene (DDT), in serum and brain were elevated in AD patients (79 control and 86 AD cases) and associated with an increased risk for AD (for the highest tertile of DDE levels, OR, 95% CI: 4.18, 2.54–5.82) [47]. On the other hand, data from the Canadian Study of Health and Aging [48] showed instead a reduced prevalence of AD (2023 participants, 399 with AD) with elevated plasma pesticide (polychlorinated biphenyls (PCB) and organochlorine (OC)) metabolite levels [49]. A more recent analysis of the cohort (513 subjects, 108 with AD) also affirmed that there is no association of PCB and OC pesticides with the risk of dementia and AD, although a posteriori analyses showed that DDE levels are related to a higher cognitive decline in time [50].

Systematic reviews and meta-analyses of reported epidemiological links have been presented [38,51,52]. A meta-analysis of past reports in 2016 (which included 3 cohort studies and 4 case-control studies) has concluded that pesticide exposure is positively associated with AD (OR, 95% CI: 1.34, 1.08–1.67) [51]. Another, more recent meta-analysis in 2019 of 19 studies of occupational exposures to multiple agents on neurodegenerative diseases (13 on AD) has also concluded that occupational exposure to pesticides increased the risk of AD (weighted relative risk (RR), 95% CI: 1.50, 0.98–2.29) [52]. A recent cohort data not included in the above meta-analysis came from the Hellenic Longitudinal Investigation of Aging and Diet study in Greece [53], which indicated an association between self-reported pesticide exposure and cognitive function [54]. Interestingly, no association between pesticides and PD could be discerned with the same cohort [55]. On the whole, therefore, results from epidemiological analyses are generally supportive of pesticide exposure being an important environmental risk for sporadic AD.

## 3. Pesticides and the Induction of Alzheimer’s Disease Markers in Cell Culture and Animal Models

Pesticides presented at high concentrations would cause acute cytotoxicity and neurotoxicity [39,40]. However, for late-onset sporadic AD, what would be of disease relevance is likely chronic, low-dose environmental/occupational exposure. In principle, pesticides could promote the onset or enhance the progression of AD via the modulation of the two major etiopathological factors—Aβ and tau. For example, the pesticide Rotenone, which inhibits the mitochondrial electron transport chain (ETC) triggers hyperphosphorylation of tau and Aβ aggregation in cultured rat neurons [56] and taupathy in rats [57]. Administration of the pyrethroid pesticide Deltamethrin, or the carbamate pesticide Carbofuran, into rats likewise triggered tau hyperphosphorylation, with activation of glycogen synthase kinase-3β (GSK-3β) and inhibition of protein phosphatase-2A (PP2A) [58]. Another pyrethroid pesticide, cypermethrin, also stimulated GSK-3β-dependent increase in Aβ and phosphor (p)-tau in rats [59]. In the same light, the organophosphate pesticide chlorpyrifos, which inhibit acetylcholinesterase [60], upregulates Aβ and tau in SN56 basal forebrain cholinergic neurons [61] and in mice [62], as well as p-tau via activation of GSK-3β [61]. DDT also increases Aβ levels in H4 glioma cells with a APP_Swe_ transgene via elevation of APP and BACE1, while decreasing the levels of Aβ-clearing ATP-binding cassette transporter A1 (ABCA1) and inhibiting the activity of Aβ-degrading insulin-degrading enzyme (IDE) [63]. Aβ production has also been shown to be elevated by triazine herbicides [64] and pyrazole insecticides in various cell lines [65].

While many reports have demonstrated pesticide-elicited neurotoxicity and AD-like pathology in terms of Aβ and p-tau, there are limitations to these models and caveats in the interpretations of findings. In several cases where experimental observations were made with relatively high doses, acute toxicity responses rather than chronic effects associated with environmentally relevant residual concentrations may have prevailed. In this regard, a recent report using residual amounts of fungicides in an AD transgenic mouse model is worth noting. Lafon et al. [66] exposed J20 mice (harboring mutant hAPP_Sw/Ind_) to a cocktail of 3 fungicides, Cyprodinil, Mepanipyrim, and Pyrimethanil, at a residual dose of 0.1 μg/L in drinking water for 9 months. This resulted in enhanced Aβ aggregation, gliosis, and neuronal loss. In the later months, the fungicides also increased vascular amyloid aggregates in a manner reminiscent of cerebral amyloid angiopathy [67,68]. The fungicides exacerbated amyloid aggregation, gliosis, and neuronal demise. Interestingly, the fungicides appear to bind to amyloid plaques and able to promote fibril formation by Aβ1-42 in vitro. Furthermore, residual fungicide elevated BACE1 protein levels while reducing that of the Aβ-degrading enzyme Neprylisin [69], although the respective transcripts did not change significantly. In another report [70] which investigated the effect of Chlorpyrifos administration into TgF344-AD transgenic rats (harboring APP_Swe_ and PS1∆E9), dosing was based on a human-derived occupational exposure paradigm established from a cohort of Egyptian agricultural workers [71,72,73]. The authors observed enhancement of cognitive impairment and behavioral deficits that is prominent only in male and not female rats, which is consistent with Chlorpyrifos’ acceleration of neurodegeneration in males. The authors did not find significant changes in amyloid and tau pathology. Instead, a persistent pathological change observed is an increase in microglia numbers and activation [70].

Taken as a whole and despite the variance in methods, models and pesticides used, both in vitro and animal-based studies have implicated a range of cellular and molecular mechanisms that could initiate or enhance AD pathology. These mechanisms shall be further discussed in the following sections.

## 4. Potential Neuropathological Mechanisms of Pesticides

**Figure 1:**
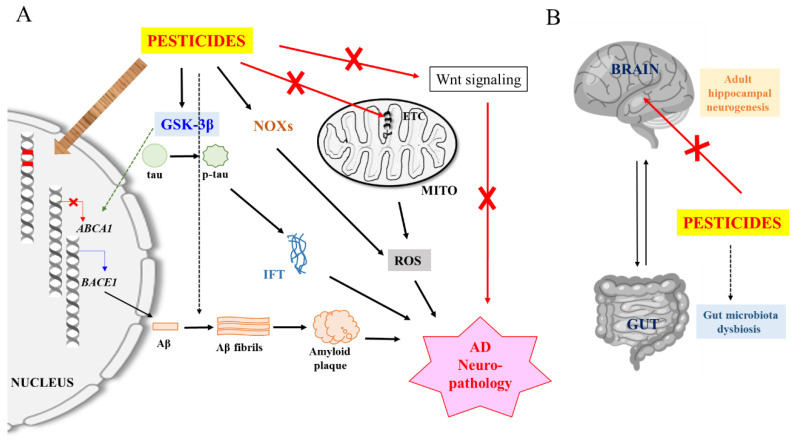
A schematic and generalized illustration of pesticide-induced Alzheimer’s disease (AD) neuropathology. (**A**) Pesticide inhibition of mitochondrial electron transport chain (ETC) components and activation of NADPH oxidases (NOXs) produce mitochondrial and neuron damaging reactive oxygen species (ROS). Damaged mitochondria and ROS could trigger neuroinflammation (not shown here for simplicity). Pesticide activation of glycogen synthase kinase-3β (GSK-3β) promotes β-site APP cleaving enzyme 1 (BACE1) expression and Aβ production while reducing Aβ clearance, and phosphorylates tau to promote intracellular fibrillary tangle (IFT) formation). Some pesticides may also promote Aβ fibril formation. Pesticides could inhibit Wnt signaling which is impaired in AD, and which reactivation improves AD disease phenotype. Pesticides and/or ROS could also cause DNA mutations and affect the expression of AD-related genes via epigenetic mechanisms. (**B**) Pesticides could inhibit hippocampal adult neurogenesis and affect AD pathology via gut microbiota dysbiosis. See text for more details.

Neurotoxic and neuropathological mechanisms underlying pesticide exposure may include the more generic detrimental processes of oxidative stress [74] and neuroinflammation [75]. On the other hand, specific Aβ- and tau-related pathways and events could also be induced or involved. These processes are heavily intertwined with Aβ production and tau phosphorylation processes in the aging brain (see Figure 1A).

### 4.1. Induction of Oxidative Stress and Neuroinflammation

Pesticides such as Rotenone are mitotoxic and inhibit the mitochondrial ETC [76]. DDT and DDE likewise impair the ETC and oxidative phosphorylation (OXPHOS) [77]. There are two important consequences to this inhibition. The first is a reduction in the product of OXPHOS, ATP. This could considerably impair the energy metabolism of cells and tissues. Altered energy metabolism has been documented in farmers exposed to pesticides [78]. Brain neurons are particularly notable in terms of energy demand [79], and impairment in ATP production via oxidative phosphorylation may lead to a metabolic shift for glucose utilization from oxidative phosphorylation to glycolysis, a metabolic reprogramming phenomenon known as aerobic glycolysis [80,81], which is prominent in the AD brain and other neurodegenerative diseases with mitochondrial impairment. This apparent survival mechanism results in lactate production, which in an APP/PS1 AD mouse model worsened cognitive performance [82]. Aβ could trigger such a metabolic switch in neurons and microglia, and for the latter it eventually leads to microglia dysfunction [83]. Interestingly, occupational-like organophosphate exposure causes microglia dysregulation [70]. In the aging brain with elevated Aβ production and accumulation, chronic low dose pesticide exposure could thus synergistically promote the impairment of both neurons and glia.

The second is an increase in reactive oxygen species (ROS), particularly superoxide, by the dysfunctional ETC, thus producing oxidative stress. Oxidative stress is an important pathological mechanism exerted by many environmental toxicants, including a large number of pesticides, such as paraquat, OCs, and organophosphates [74,84]. This is particularly so when some pesticides (eg., paraquat, DDE and Chlorpyrifos) could also induce the Nicotinamide adenine dinucleotide phosphate (NADPH) oxidases [85,86,87], which acute release of ROS could cause neuronal death and degeneration [88]. Damaged and ROS-generating mitochondria trigger an inflammatory response initiated by the NACHT, LRR and PYD domains-containing protein 3 (NLRP3) inflammasome, particularly in microglia [89,90,91,92]. The resulting production of the pro-inflammatory cytokine IL-1β impairs neuronal health and function. AD is known as a disease of chronic systemic inflammation, which is a result of contributions from many factors [93] that would include pesticides.

### 4.2. Enhancement of Aβ and tau Expression, Modification and Clearance

A range of pesticides may act in a manner that is termed by Cam and colleagues as environmental “Alzheimerogens’ [65], namely by elevating the levels of Aβ [59,61,62,63,64,65,66]. The authors screened a large compound library using a cell-based assay for enhanced production of the longer Aβ peptides (Aβ42/Aβ43), and identified 9 pyrazole insecticides. These were found to induce, in a β- and γ-secretase-dependent manner, an increase in extracellular Aβ42 in various cell lines and neurons differentiated from induced pluripotent stem cells (iPSCs) derived from healthy and familial AD (FAD) patients. Pesticide induction of Aβ usually occurs as a consequence of increases in the expression or activity of BACE1 and γ-secretase [59,63,65,66], often in combination with a reduction in Aβ clearance due to a suppression of the levels of Aβ-degrading enzymes such as IDE or neprylisin [62,63,66]. These pesticides would often also induce an increase in the levels of tau and p-tau [57,58,59,61,94].

What is the underlying mechanism(s) for the elevation of Aβ and p-tau by pesticides? An important central regulator of these pathological processes appears to be GSK-3β, which is one of two GSK isoforms (GSK-3α and GSK-3β) that is widely expressed, up-regulated in the aging brain [95], and is a critical pathological factor in AD [96,97]. GSK-3β is constitutively active with Tyr216 autophosphorylated, but could be inhibited by phosphorylation at Ser9 via AKT kinase, and as such could in turn be reactivated by protein phosphatase 2A (PP2A) dephosphorylation of Ser9 [98]. GSK-3β regulates BACE1 expression in a nuclear factor κB (NFκB)-dependent manner. In the 20E2 line expressing human Swedish mutant APP, GSK inhibition by its inhibitor AR-A 014418 reduced Aβ production, and this is due to GSK-3β’s (but not GSK-3α) regulation of the *BACE1* gene promoter via the activation of NFκB [99] GSK-3β also modulates the localization of PS1 [100], which is one of its substrates. Importantly, GSK-3β is also a major tau kinase [98,101,102]. Rotenone activates GSK-3β by enhancing its phosphorylation at Tyr216 while inhibiting phosphorylation at Ser9 [103], and Rotenone-induced cytotoxicity could be attributed to microtubule destabilization caused by reduction in the binding capacity of p-tau [104]. While the mechanistic aspects of how pesticides of different classes activate GSK-3β have not been thoroughly investigated, it is clear that increased GSK-3β activity likely underlies the Aβ and p-tau pathology exerted by most pesticides. Another interesting point to note is that Aβ and p-tau pathology are interconnected, and one such connection occurs in the form of Aβ42’s stimulation of tau hyperphosphorylation through its interaction with GSK-3α [105].

A further potential connection with regards to the above is pesticides’ potential suppression of the Wnt/β-catenin signaling pathway [106,107,108]. Paraquat exposure altered the levels of the Wnt pathway genes in mouse neural progenitor cells [106], rotenone impaired Wnt signaling in a Drosophila PD model [107] and Deltamethrin also reduced Wnt signaling pathway genes in zebrafish’s development of swim bladder [108]. Wnt signaling is known to be impaired in AD [109,110,111], and activation of the Wnt/β-catenin signaling pathway represses BACE-1 expression [112]. Activation of Wnt signaling has been shown to rescue memory loss and improves synaptic dysfunction in APP/PS1 AD transgenic mice [113,114]. In the well-known canonical Wnt signaling pathway, Wnt’s binding to its receptor Frizzled and the downstream processes leading to the stabilization of β-catenin involves the inhibition of GSK-3β [115]. It is conceivable that some pesticides could also inhibit Wnt signaling through their action on the Wnt receptor Frizzled like the anti-helminthic drug Niclosamide [116], but this is yet unclear.

### 4.3. Promotion of Amyloidogenesis

Beyond Aβ production, pesticides could also potentially promote aspects of amyloidogenesis, such as Aβ oligomerization and fibril formation, as well as amyloid plaque formation. Evidence in this regard are scarce, but this possibility has been recently demonstrated by Lafon et al. [66], in which the fungicide cocktail of cyprodinil, mepanipyrim, and pyrimethanil associate with amyloid plaques and appears to promote Aβ fibril formation in vitro, as described in more detail in Section 3 above. Rotenone exposure (as low as 0.5 nM) of neuron cultures from rat hippocampus, substantia nigra and locus coeruleus resulted in the formation of protein aggregates of α-Synuclein and Aβ [56]. Aβ peptides are known to trigger aggregation of α-Synuclein [117], and there is a possibility that heterotypic amyloid co-aggregates could be formed via a α-Synuclein seeding mechanism [118]. Whether such co-aggregations could actually be triggered by any pesticide in cultured neurons or in animal brain remain to be demonstrated.

## 5. Speculative Neuropathological Mechanisms of Pesticides

The section above has outlined neurotoxic and neuropathological mechanisms of pesticides that have been clearly demonstrated in various experimental models, and for which there are empirical support. In this section, pesticide-associated AD etiological mechanisms that are more speculative and indirect in nature shall be highlighted (see Figure 1A,B).

### 5.1. DNA Damage and Somatic Mutations

Pesticides such as OCs are known to be genotoxic and induce DNA damage [119,120], either via oxidative damage [121] or direct interaction [122] with DNA. Pesticide genotoxicity is usually considered in the context of their oncogenic potential [123,124], particularly during episodes of acute exposure with mutational damage to dividing cells, such as neural stem cells (NSCs) and neural progenitor cells (NPCs). However, chronic low dose exposure may also result in non-lethal mutations that occur cumulatively in terminally differentiated, non-dividing neurons. These mutations could impair neuronal health and function in largely undefined ways. Interestingly, somatic mutations or variants in brain neurons with low allele frequency has been associated with aging and neurodegenerative diseases [125,126,127]. This somatic mosaicism has, in particular, been demonstrated in AD brains. *APP* variants have been shown to occur in human neurons mosaically as thousands of intronless variant ‘genomic cDNAs’ [128]. Loss of function mutation in the Peptidyl-prolyl cis-trans isomerase NIMA-interacting 1 (PIN1) [129], which is important for AD etiology, as well as signaling pathway genes that contribute to hyperphosphorylation of tau, have also been identified in AD brains [130]. It is therefore conceivable that low frequency mutations or variance generated by chronic genotoxicity of pesticides could contribute to the etiology of sporadic AD.

### 5.2. Epigenetic Mechanisms

Beyond changes to DNA sequences, pesticides could affect gene expression epigenetically [131,132]. There is evidence that pesticide exposure is linked to alterations in DNA methylation [133,134,135]. Of particular relevance in this regard would be genes that encode proteins affecting AD etiology, such as that encoding Paraoxonase-1 (PON1) [136,137]. Pesticides are also known to affect the expression of micro(mi)RNAs [138,139,140]. In particular, Paraquat’s modulate miRNAs affecting components of Wnt signaling [138] would be of direct relevance to AD [110,113].

### 5.3. Effect on Adult Neurogenesis

Pesticide exposure has been extensively linked to developmental defects [141], and is known to affect neural stem cells and neural progenitor cells in the pre- and postnatal periods [142,143]. While early life exposure to pesticides affect both neurons and glia, and impairs learning and memory [144], pesticides could also potentially impact on the arguably more AD-relevant process of adult neurogenesis, particularly at the hippocampal neurogenic sites [145]. Adult neurogenesis at the subventricular zone (SVZ) of the lateral ventricles and the subgranular zone of the dentate gyrus (DG) in the hippocampus has been demonstrated extensively in rodent animal models, and is functionally linked to memory encoding as well as behavioral modification [145,146]. In this regard, neonatal exposure to permethrin pesticide in mouse has been shown to cause lifelong fear and spatial learning deficits and alters hippocampal morphology of synapses [147]. Repeated pyrethroid exposure of adult mouse also causes hippocampal endoplasmic reticulum (ER) stress and learning deficits [148].

Of particular relevance here is that adult neurogenesis is linked to AD [149,150], and impaired adult neurogenesis could be an early event in AD [151]. Conceivably, neuronal vulnerability to AD etiology may be exacerbated by earlier defects in the progenitor cells, while adult neurogenesis could be a compensatory response to neuronal loss to the disease condition. A recent comparative analysis of human dentate gyrus samples indicated that while hippocampal neurogenesis is prominent in normal adults, this is drastically reduced in AD patient brains [152]. Impairment of adult neurogenesis specifically exacerbates AD neuropathology in APPswe/PS1ΔE9 AD trangenic mice [150]. In this connection, Paraquat has been specifically shown to inhibit hippocampal neurogenesis in adult mice, as intraperitoneal administration of the compound for 3 weeks inhibited neural progenitor cell proliferation, altered developmental fate of newly generated cells in the hippocampus and impaired hippocampus-dependent learning and memory [153]. Perinatal exposure of the herbicide glufosinate-ammonium (GLA) to pregnant mothers inhibits SVG neurogenesis in newborn mice and altered the neuro-glial differentiation of cultured mouse primary neural stem cells [154,155]. Given the above connections between pesticides, adult neurogenesis and AD, it could be reasonably deduced that pesticide disruption or inhibition of adult neurogenesis may initiate or otherwise promote AD pathology.

### 5.4. Dysfunction of the Brain-Gut Axis

Brain health is influenced by the health of the gut, or more specifically the gut microbiome [156]. The two-way communication between gut microbiota and the brain through the enteric nervous system, the vagus nerve, and the immune system, involving tryptophan metabolism and microbial products, constitutes what is often termed the microbiota-gut-brain axis [157]. The gut microbiota modulate neuroinflammation, and would therefore have role in AD [158,159]. Gut microbiota dysbiosis is known to occur in AD [160], and its induction could aggravate disease progression in AD models [161]. Evidence for this notion is provided by, for example, a recent report using an AD-like pathology with amyloid and neurofibrillary tangles (ADLPAPT) transgenic mouse model of AD [162], whereby transplantation of fecal microbiota from wild-type mice into ADLPAPT mice ameliorated plaque and NFT formation, reduced glial activation, and alleviated cognitive impairment [163]. A number of reports have now indicated that a wide range of pesticides, such as the fungicide propamocarb [164], glyphosate herbicides [165], carbamate insecticide Aldicarb [166], and Chlorpyrifos [167,168] are all known to alter gut microbiota and cause varying degrees of dysbiosis. Exposure of propamocarb to mice at 3–300 mg/L through drinking water for a duration of 28 days changed the microbiota in the cecal and fecal contents at phylum or genus levels [164]. Sub-chronic and chronic exposure of mice to glyphosphates increased anxiety and depression-like behaviors and altered the gut microbiota composition, decreasing the *Corynebacterium, Firmicutes, Bacteroidetes* and *Lactobacillus* in particular [165]. Analysis of low dose Chlorpyrifos exposure at the late postnatal pre-weaning stage of rats indicated changes in neurotransmission parameters and induction of gut microbiota dysbiosis at both genus and species levels [167]. Given these connections, it is thus conceivable that pesticide-induced gut microbiota dysbiosis could play at least an indirect role in AD pathogenesis.

## 6. Epilogue

In the paragraphs above, recent works on the connection between pesticide exposure and AD were highlighted and discussed. Epidemiological studies are increasingly affirming an association between environmental and occupational pesticide exposure and AD. Laboratory experiments have also better defined the potential neurotoxic and neuropathological mechanisms induced by pesticides that might be AD-initiating or -promoting. The more classical and generic mechanisms would include chronic oxidative stress, neuroinflammation, and Aβ/p-tau neuropathology, which impact negatively on aging neurons. Beyond these, there are more speculative or indirect mechanisms, which include somatic mutation, epigenetic modulations, adult neurogenesis impairment, and gut microbiota dysbiosis. It is clear that these mechanisms crosstalk extensively with the classically-perceived mechanisms, and the neuropathology of AD is a tangled web of factors, pathways and consequences that often mutually reinforce and synergize. The speculative/indirect mechanisms further attest to the common notion that sporadic AD is etiologically complex. However, from the perspective of a group of environmental risk factor like pesticides, key components of the entangled web of neuropathological mechanisms in AD that may be therapeutically relevant and useful could also be effectively highlighted, and thus potentially exploited.
